# “They test my blood to know how much blood is in my body”: the untapped potential of promoting viral load literacy to support adherence and viral suppression among adolescents living with HIV

**DOI:** 10.1002/jia2.26153

**Published:** 2023-10-25

**Authors:** Sarah Bernays, Joni Lariat, Frances Cowan, Beula Senzanje, Nicola Willis, Zivai Mupambireyi Nenguke

**Affiliations:** ^1^ School of Public Health University of Sydney Sydney New South Wales Australia; ^2^ Department of Global Health and Development London School of Hygiene & Tropical Medicine London UK; ^3^ Liverpool School of Tropical Medicine Liverpool UK; ^4^ UNICEF Harare Zimbabwe; ^5^ Zvandiri Harare Zimbabwe; ^6^ Centre for Sexual Health HIV/AIDS Research (CeSHHAR) Harare Zimbabwe

**Keywords:** adolescents, HIV, antiretroviral therapy, adherence, viral load literacy, viral suppression

## Abstract

**Introduction:**

Achieving sustained HIV viral suppression is a key strategy to optimize the health and wellbeing of those living with HIV. Sub‐optimal adherence to antiretroviral therapy (ART) in adolescents and young people living with HIV (AYPLHIV) in Southern Africa, due to a range of social and contextual factors, including poor mental health, has presented a substantial challenge to meeting targets aimed towards improving treatment outcomes and reducing transmission. With the increasing availability of viral load (VL) testing in Southern Africa, there is an opportunity to better understand the relationship between VL literacy, wellbeing and adherence among adolescents.

**Methods:**

We conducted qualitative interviews with 45 AYPLHIV aged 10–24 years in three districts (urban, peri‐urban and rural) in Zimbabwe between March and August 2021. The sample was purposively selected to represent a range of experiences related to HIV status disclosure, gender, marital status and treatment experience. Separate workshops were conducted with 18 healthcare workers (HCWs) and 20 caregivers to better understand existing support mechanisms to AYPLHIV accessing ART. We used thematic analysis to examine adolescent VL literacy, treatment support networks, experiences of clinic interactions, VL testing procedures and barriers to adherence.

**Results:**

VL literacy was consistently under‐developed among participants. Comprehension of phrases commonly heard during clinic visits, such as TND (target not detected) and “high” and “low” VL, were better understood by older participants. VL testing was predominantly understood as a clinical procedure that enables HCWs to monitor treatment adherence. Absent throughout the interviews were descriptions of how viral suppression improves health and quality of life, likely fosters wellbeing and enhances self‐esteem, enables participation in education and social activities, and eliminates the risk of onward transmission.

**Conclusions:**

It is imperative that we reconsider how routine VL monitoring is communicated to and understood by AYPLHIV. Reframing ART, including VL test results, in terms of the psychosocial benefits that viral suppression can generate is likely to be crucial to motivating AYPLHIV to maintain optimal treatment engagement and develop self‐management approaches as they move into adulthood. Access to accurate information tailored to individual concerns and circumstances can support AYPLHIV to achieve wellbeing.

## 1 INTRODUCTION

Adolescents and young people living with HIV (AYPLHIV) continue to have the worst health outcomes of all age groups living with HIV [1]. Rates of viral suppression remain lower in this group than in children and adults, due to higher rates of attrition from care and suboptimal adherence to antiretroviral therapies (ARTs) [2, 3, 4, 5, 6, 7, 8].

The usual stressors associated with adolescence that place adolescents and young people at higher risk of mental health conditions [9] are exacerbated by the additional challenges AYPLHIV face during this period [9, 10, 11]. This can significantly impact treatment adherence, leading to inconsistent viral load (VL) and low rates of viral suppression [9, 12, 13, 14, 15, 16]. Adherence and retention in care has also been shown to be impacted by structural factors associated with poverty [17]; social stigma [2, 3, 18]; relationships with healthcare workers (HCWs) and caregivers [19, 20]; and, the transition from adolescent to adult HIV care and from caregiver‐supported to autonomous treatment taking [21]. These factors are interconnected and are exacerbated by the limited opportunities available to address mental health challenges arising from growing up with HIV, particularly in resource‐constrained settings, such as in Southern Africa where the epidemic has been concentrated [9]. Significant attention has been invested in supporting adherence interventions [22] with variable success and patchy coverage [22, 23]. However, a pattern has emerged that the most effective interventions in improving adherence primarily focus on providing psychosocial care, commonly delivered by peers, to support AYPLHIV's wellbeing [24, 25, 26, 27, 28, 29, 30].

Routine VL testing is a critical component in improving treatment opportunities for HIV in resource‐constrained settings, and is a particularly useful tool among groups with a high risk of virological failure, such as adolescents [31], to identify how to target enhanced support [6].

Adolescents often have poor HIV literacy, particularly in low‐and‐middle‐income settings where resources are limited [32, 33]. As VL monitoring has become more widely available in most settings, VL literacy, that is understanding the meaning of VL test results and the broader implications of these results for health and wellbeing, could become a core component of HIV literacy. For example, understanding Undetectable equals Untransmittable (U = U) and Treatment as Prevention (TasP) has changed the narrative about what it means to live with HIV and is the basis for much of the optimism around the global targets aiming to “eliminate” AIDS. VL literacy is having a positive effect on stigma reduction and improving wellbeing among adults who are living with HIV [34, 35]. However, VL literacy among AYPLHIV has received little attention. Significantly, the emphasis on adherence behaviour among adolescents in the literature remains orientated towards protecting against ill health rather than optimizing good health and quality of life [36].

Given how integral viral suppression is to current 95‐95‐95 global targets, particularly among the most vulnerable and at risk groups, it is important to understand how AYPLHIV understand VL results, how this influences their treatment engagement and the potential value of investing in its development. In this paper, we examine how AYPLHIV understand VL monitoring and consider how improved VL literacy might impact upon the interconnected spheres of AYPLHIV wellbeing, adherence and viral suppression.

## 2 METHODS

### 2.1 Study design and sampling

This paper draws on qualitative data collected through individual in‐depth interviews with 45 AYPLHIV aged 10–24 years and six participatory workshops conducted with 20 caregivers and 18 HCWs between March 2021 and August 2021. Participants were recruited in three healthcare facilities from high HIV burden districts in Zimbabwe: Harare (Hopley), Bulilima and Buhera districts; representing a rural primary care facility, an urban primary care facility and a peri‐urban secondary care facility. Forty‐five AYPLHIV out of 63 eligible individuals were selected from facility ART registers (15/site), using purposive sampling with the aim of maximum variation, considering age, gender, duration on ART, current ART regimen (first/second line), timing of last VL test and mode of transmission. All participants were fully aware of their HIV status prior to enrolment in the study.

### 2.2 Data collection

Individual semi‐structured interviews with AYPLHIV, conducted by local, trained social scientists, explored their experiences of VL testing, understanding of viral suppression and its influence on treatment engagement and wellbeing. Three participatory workshops (one/site) were conducted with 20 purposively selected caregivers (aged 39–64 years) connected to AYPLHIV and three participatory workshops with 18 HCWs (aged 34–54 years) who directly provided HIV treatment and care services to AYPLHIV. Conducted by the same team of local, trained social scientists, these workshops explored their perspectives on improving adolescent adherence, including how VL monitoring could be better communicated to adolescents. To maximize participation, we used participatory tools, such as voting and topic cards.

### 2.3 Data analysis

All the data were audio‐recorded, transcribed verbatim and translated from the local languages of Shona and Ndebele into English for analysis. A coding framework was developed by SB and JL through independent line‐by‐line coding. The coding framework was finalized through consensus and then applied to all remaining transcripts to conduct thematic analysis. Themes and patterns were identified by triangulating the data, to support insights into AYPLHIV's lived experiences within the broader relational context which framed their understanding of VL literacy.

### 2.4 Ethics

The Ministry of Health and Child Care (Zimbabwe) approved the study and ethical permission was granted by the Medical Research Council of Zimbabwe (MRCZ/A/2657). Written informed consent/assent were obtained from all participants. Parental informed consent was sought for all adolescents below the age 18 years from primary caregivers. Pseudonyms have been used in this paper to protect anonymity.

## 3 RESULTS

Forty‐five AYPLHIV aged 10–24 years were recruited. The sample characteristics are outlined in Table 1. All participants were attending community‐based psychosocial support groups.

**Table 1:** Characteristics of AYPLHIV study participants (*n* = 45)

District	Buhera	Bulilima	Hopley
Characteristics			
**Age**			
10–14 years	3	3	5
15–19 years	6	6	6
20–24 years	6	6	4
**Orphanhood status**			
Double orphans	4	7	5
Maternal/paternal	5	5	7
Non‐orphans	6	3	3
**ART**			
First line	12	13	10
Second line	3	2	5
**Marital status**			
Married	4	1	2
Single	11	14	13

*Note*: The sample characteristics of the 20 caregivers (aged 39–64 years) and 18 healthcare workers (aged 34–54 years) are outlined in Table 2 and Table 3, respectively.

**Table 2:** Characteristics of caregiver study participants (*n* = 20)

Caregiver (*n* = 20)	
Characteristics	
**Sex**	
Male	3
Female	17
**Marital status**	
Married	10
Single	4
Widowed	6
**Employment status**	
Formally employed	4
Informally employed	5
Unemployed	11
**Caregiver type**	
Biological parent	12
Non‐biological primary caregiver	8

**Table 3:** Characteristics of healthcare worker study participants (*n* = 18)

Healthcare worker (*n* = 18)	
Characteristics	
**Sex**	
Male	3
Female	15
**Employment position**	
Opportunistic infection nurse	10
Primary care counsellor	8

### 3.1 VL literacy

Most adolescents understood that HCWs used VL testing to monitor adherence and identify problematic adherence behaviour. There was little evidence to suggest that AYPLHIV understood how viral suppression might positively impact their social and relational lives.

Participants had, at the minimum, a basic knowledge about how HIV impacts the body and the importance of ART. Many drew on well‐developed metaphors, such as describing CD4 cells as soldiers, to explain the interaction between ART and the virus. Most younger participants (10–14 years old) had only a limited understanding of what their VL results meant. Older adolescents (aged 16–24) typically demonstrated a greater understanding of how VL results provide information about their body's immunological response to the virus and their treatment.

“On viral load they will be checking if you are taking your medication correctly and if you are not defaulting. They will be checking to see how many soldiers [CD4 cells] are in your body and if they can suppress your viral load.” (Susan, 23‐year‐old female)

Many older adolescents (16+) were able to explain the clinical objective of VL testing:

“When they are testing your viral load, the reason is to see how many red blood cells you have and how much virus you have in your blood and whether it is going up or going down or it's stagnant.” (Richard, 24‐year‐old male)

Although HCWs reported providing age‐appropriate immunological explanations when introducing VL testing, younger adolescents were rarely able to confidently recount them during our interviews. Several participants reported that they could not remember ever being told why their blood was being tested and they did not know what VL testing meant.

Consistently, the primary reason that AYPLHIV gave for why blood was being taken, was to allow HCWs to infer whether they were taking their treatment as “they should.” Even among those who were familiar with the term “suppressed” or “undetectable,” VL testing was defined as a proxy for assessing adherence behaviour.

Interviewer: Alright so how does having a suppressed viral load help you?

“Participant: It helps because I would have followed all the instructions properly.” (Thembinkosi, 21‐year‐old female)

Across all age and gender groups, further understanding about VL, such as what an undetectable VL means and how viral suppression prevents onward transmission, was limited. Only one participant, a 24‐year‐old male trained as a CATS in an HIV peer‐support programme, was able to describe the relationship between being undetectable and transmissibility:

“Having an undetectable viral load helps in that if you are a guy, and you have a girl, if you have sex your chances of infecting her are low.” (Richard, 24‐year‐old male)

### 3.2 Missed opportunities to develop VL literacy

Throughout our interviews, a pattern emerged of missed opportunities during the process of VL testing to explain the procedure taking place, or to enhance or reinforce clinical knowledge about HIV, ART and VL testing, and critically to foreground the psychosocial benefits of achieving a low or undetectable VL. There were no reported examples of nursing staff utilizing the interaction to reinforce existing knowledge or develop new understandings that might incentivize optimal adherence.

HCWs explained the constraints that limit their ability to engage consistently in detailed conversations about VL test results with all the AYPLHIV in their care.

“We see a lot of children in a day and it's very difficult to remember when the VL was taken and if the results were disseminated or not, especially for those with a suppressed viral load. Remember we are overwhelmed, and we will be in a rush to serve other clients. If parents ask for the results, they will be shown.” (35‐year‐old female nurse).

As illustrated, HCWs must triage their clients according to need. Those with a suppressed VL who are not in immediate need of referral to counselling will often receive less direct attention from nursing staff because they were not considered in need of “enhanced support.”

Caregivers primarily characterized their role in their adolescent's HIV treatment as being one of treatment supervision and encouragement. None suggested that they explained VL test results beyond whether they were low or high or the broader implications of viral suppression.

“Our job as parents is to encourage them to take their medication correctly without skipping and taking it on time so that their viral load will remain low and taking balanced diet meals. We also encourage them if they are dating to use protection so that they will not spread the virus and protect their partners” (Luyanda's mother, 45 years old).

For many caregivers, encouragement extended to various aspects of everyday life, including diet and relationships. This shows that discussing sex, in the context of HIV prevention, is not necessarily a silenced or avoided topic. It also shows that the link between aiming for sustained viral suppression and the risk of onward transmission is not fully explored for its motivating potential. This could be because caregivers, particularly those who are HIV seronegative, are also unaware of this information.

### 3.3 Deductive interpretation of results

Whether adolescents became aware of their test results was variable but appeared to depend largely on their age and VL result. For those who returned a low or undetectable VL, results were noted in their medical file and tended not to be discussed with them. Some older participants reported taking the initiative to independently check their results. However, without having had prior opportunities to develop their understanding of what the results could mean and in the absence of any guided interpretation, it remained just a number which held little interpretive value.

All participants assumed that the absence of any intervention or discussion with staff about their test results indicated that no adjustments were needed and that there was no cause for immediate concern. The opportunity to provide direct reassurance about what could be deduced from an undetectable result or to further incentivize continued adherence went unrealized.

### 3.4 Caregivers’ engagement with VL results: no news is good news

Caregivers described a strong sense of responsibility to ensure their child's optimal adherence, especially among those with children under 15 years old. However, for many, accompanying their child to clinic appointments to check on their adherence and health was rarely feasible given their other income‐generating and/or additional caring commitments. Several caregivers of older adolescents, who are encouraged to attend independently, said that they often felt frustrated by the lack of information they received from their child and the inconsistent contact they had with HCWs. It was common that they would get information inadvertently when the clinic called searching for their child who may have missed a review or had received a high VL test result.

Even among the caregivers who were able to attend the clinic with their child, this rarely facilitated access to personalized information about their child's state of health, with minimal discussion of their child's results within these clinic reviews. Their limited engagement with VL monitoring followed a similar pattern to that observed among adolescents, in which they assumed that an absence of action (and related discussion) by HCWs could be interpreted as a positive signal of their child's health.

We found that caregivers’ VL literacy was also limited, following a similarly narrow pattern to young people in which VL testing should serve only to monitor adherence behaviour. As 19‐year‐old Sandile explained, “my mother receives my results and then she encourages me to continue taking the medication because my life depends on it.” For ethical reasons, caregivers were not asked to reveal their HIV status, but given at least some were their child's biological parent, it is reasonable to assume that some may have also had a positive HIV status, suggesting that they may not have been aware of (or willing to reveal) a fuller understanding of the implications of viral suppression.

### 3.5 A clinical gauge of behaviour: reinforcing binary categories of good or “failing” adherence

The categorization of test results as either “high” (above 1000 copies/ml) and “low” (below 1000 copies/ml) VL enabled HCWs to quickly determine a patient's clinical care requirements. A high result triggered an alternative course of action: increasing the frequency of clinic visits with treatment given only for 1 month at a time, rather than for 3 months, and a referral for enhanced adherence counselling.

Many adolescents considered that their relationships with HCWs hinged on the return of a “low” test result. An 11‐year‐old boy, Edward, said that “I get along with the nurses because I follow their instructions and I take my medicine on time,” suggesting that a smooth relationship depends upon demonstrating (through VL test results) good adherence behaviour. Young people anticipated that HCWs would interpret a “high” VL, which was assumed to be a proxy for non‐compliance, as reflecting disobedient or troublesome behaviour, thus negatively impacting their relationship. Given caregivers’ sense of responsibility for ensuring adherence, framing VL results as reflective of behaviour may have had wider repercussions, further entrenching secrecy about the drivers of precarious adherence. It was not implied or explicitly stated that a bad experience with an HCW influenced non‐attendance. Given the lack of alternatives and that young people may not recognize that the care they receive could be different, we would expect that they would still attend but might be even more reluctant to share their concerns or questions.

Occasionally, however, participants expressed that monitoring would facilitate access to help for challenges they are facing, and support HCWs to recognize potential indicators of poor wellbeing not otherwise identified through discussion, as exemplified in the following quote:

Viral load testing means that I can be helped on time. They (HCWs) say that if I don't get tested there is no way they are going to be able to help me. Let's say I don't get tested. Maybe I am not taking my medication well, I just have them at home, then I don't get tested so there is no way they are going to know what is happening to me. (Richard, 24‐year‐old male)

The singular focus on rectifying “poor” adherence, without also reinforcing good adherence behaviours or offering optimistic narratives associated with viral suppression, appeared to leave some participants resistant to engage with available support. They considered that VL monitoring risked punitive consequences, reinforcing the limited conceptualization of VL testing as serving to identify, and potentially “catch out,” those who are struggling.

## 4 DISCUSSION

This study revealed low levels of VL literacy among participants, indicating that VL monitoring is under‐utilized in the treatment and support of AYPLHIV. Participants in this study were all attending support groups providing psychosocial support for AYPLHIV and so are likely to have better VL literacy than those who are not engaged in support programmes, suggesting that low levels of VL literacy is likely to be a widespread problem. This is reinforced by the relative absence of any focus on VL literacy for AYPLHIV within the literature [36].

This study also supports what is already well‐documented in the literature: that when discussions do occur, they tend to focus on mitigating the negative outcomes of suboptimal adherence [21, 37, 38], and overcoming adherence barriers through practical strategies, such as developing a better routine, reinforcing the need for parental supervision or disciplining what are perceived to be negative attitudes towards treatment [10, 21, 39]. Rarely, if ever, was VL testing harnessed as an opportunity to explain the benefits of viral suppression for AYPLHIV's social and relational lives. This was clear in the consistent silence that immediately followed interviewer's invitation for AYPLHIV participants to share their understanding of viral suppression and what an undetectable VL means. It was also clear in how caregivers and HCWs described their roles in supporting AYPLHIV.

Currently, VL monitoring is used to assess progress towards global and national targets, or to identify adherence behaviour issues at the individual level. Limiting the use of test results in this way forgoes a critical opportunity for it to simultaneously be used as a social tool to build health literacy and motivation among AYPLHIV, as well as to support differentiated tailored support at an individual level. Furthermore, by perpetuating a false binary that adherence falls into two discreet categories (good/bad), current efforts fail to reflect that achieving and sustaining viral suppression is challenging and likely to be an imperfect journey. We propose that this narrow framing misses out on building VL literacy by centring the psychosocial benefits of viral suppression in conversations between HCWs, adolescents and their caregivers may incentivize adherence, which in turn may support positive outcomes for wellbeing.

### 4.1 More than a number: translating viral suppression into socially enabling narratives

The untapped potential of VL literacy noted in our study reflects a trend identified in a recent World Health Organization (WHO) global consultation with AYPLHIV, which found very uneven VL literacy among young people [40]. This gap between assumed and actual knowledge may explain why the need for investment in conversations to generate and sustain a widespread understanding of the positive implications of viral suppression remains under‐recognized. VL literacy can enable test results to be interpreted as more than a number reflecting good or failing adherence and can equip adolescents to appreciate the value of their adherence more broadly. It can stave off complacency and motivate continued adherence because of the proof that it provides that their treatment is working. This is critical given that research with young people has indicated that in the absence of accurate information about how ART works, feeling healthy and not experiencing symptoms can appear to justify stopping treatment [39].

There is growing evidence that integrating person‐centred psychosocial support and HIV clinical care has the potential to significantly improve wellbeing among PLHIV [13, 14, 41, 42, 43]. The evidence also suggests that improved wellbeing can facilitate consistent adherence and increase the likelihood of sustained viral suppression [13, 14]. Our findings, and that of the WHO global consultation [40], suggest that conveying VL test results in ways that are individually meaningful may allow adolescents to appreciate the relevance of their results and this may, in turn, support improved adherence. AYPLHIV in this study highlighted the significance of relationships with family and friends, future intimate relationships, avoiding stigma, looking healthy and having an ordinary life as key barriers and motivators for maintaining optimal adherence. Translating VL test results from a number to a story that extends beyond the clinic into young peoples’ lives sparks the potential of VL to radically transform young people's relationship with their health and treatment.

Ross and colleagues’ five domains of adolescent wellbeing is useful for considering the opportunities facilitated by developing VL literacy among AYPLHIV [44]. The framework describes the multidimensionality of adolescent wellbeing and the support needed to achieve wellbeing within each domain. It encompasses the subjective, objective and relational constructs of wellbeing, and so includes feelings of optimism and fulfilment, access to material resources, such as income, food, housing education and social networks, and harmony across one's personal, societal and environmental relationships [44]. The framework's emphasis on adolescents’ right to information, care and services to promote agency and resilience is particularly relevant to what VL literacy may enable for AYPLHIV.

Although our findings highlight its absence, drawing on the emphasis participants’ placed on healthy relationships, we propose that conversations with HCWs and knowledgeable caregivers who explain the clinical meanings of VL results through the social benefits that viral suppression facilitates are likely to enable young people to appreciate how their health and wellbeing are interconnected. Our hypothesis that there is considerable untapped value in VL literacy if it aims to motivate and build self‐esteem and hope is supported by its alignment with the key dimensions of sustained and supported adolescent wellbeing described by Ross and colleagues [44]. Using VL testing to connect viral suppression to these desired outcomes and grounding them in conversations about their interests, hobbies, values, worries and hopes for the future is likely to enhance the value of VL testing by presenting it in ways that appear to be meaningful and consequential for young people. For example, a key insight from the WHO global consultation [40] was that emphasizing the health and social benefits associated with sustained viral suppression radically improved mental health and wellbeing. Benefits such as increased energy levels, improved physical appearance, enhanced capacity to engage in educational, employment and social activities, and the possibility that the risk of onward transmission (to a partner or future child) can be eliminated were described by participants in the consultation as truly motivating. We suggest that these are the very supports adolescents need to achieve the social connectedness, agency and resilience that are the basis for optimal wellbeing.

Facilitating this shift towards improved VL literacy requires recognizing and investing in VL literacy as a central component in HIV support and counselling for all PLHIV, including adolescents and young people [36]. It is well documented, and supported by HCWs accounts in this study, that HCWs’ lack the time, training, access to tailored resources and guidelines to be able to prioritize talking with young people about their health, whether it be HIV status, ART or VL literacy [45, 46]. Developing HCWs’ communication competency, confidence and access to tailored resources and guidelines that emphasize the need to prioritize talking with AYPLHIV about their health will be required. While tailored to the developmental stage, VL literacy should be integrated into the process of disclosure, so that the relationship between adherence, viral suppression and wellbeing is introduced from the earliest opportunity. We need to create space and time for these intentional conversations to occur, given that finding such opportunities within existing care are becoming more challenging as efforts focused on fast‐tracking clinical care and reducing HCWs’ engagement with clients at health facilities are stepped up. Once initiated, opportunities for further reinforcing discussion could be cascaded throughout the psychosocial support AYPLHIV receive as part of their care. For example, the promotion of VL literacy could be supported by trained, mentored peer counsellors within the clinic or outside of healthcare facilities, who can be part of providing psychosocial support as integral to standard care for AYPLHIV.

Our research has contributed to the development of resources to support this shift in practice. We developed a VL literacy package for AYPLHIV which included two animations “Not just a number: Understanding your viral load” and “Taking charge of HIV: The journey to undetectable” [47, 48] and accompanying discussion guides to be disseminated by the peer‐led HIV and mental health programme, Zvandiri, as online resources. To ensure the relevancy and fidelity of our findings, we infused the personal narratives of the fictional characters with the words, experiences and aspirations of the young people who participated in this study. These are yet to be evaluated, but anecdotally have been useful tools for CATS to describe VL in their interactions with AYPLHIV.

### 4.2 Taking “perfect use” off the table: reframing adherence as precarious and always in need of responsive support

Research has shown that adherence can not be understood as detached from the social and relational lives of those engaging in ART, but is an active practice that waxes and wanes in accordance with shifts in motivation and competing burdens and responsibilities [21, 39, 49]. Shifting focus from “non‐adherent” or “failing” adherence, by taking “perfect use” off the table and situating VL test results within an individual's treatment journey could improve AYPLHIVs’ relationships with HCWs and motivate consistent engagement [10, 50, 51]. AYPLHIVs’ understandings of “health, illness, and medicines‐taking” is in flux; it is in a constant process of re‐definition, often experienced in tension with the production of those very themes at home, in the clinic and in the broader social contexts in which their lives are lived. “Doing adherence” better captures the lived reality of what it means for AYPLHIV to engage in long‐term ART because it acknowledges the necessary everyday work required to constantly and consistently reinstate ones’ treatment practices in such flux [52].

To maximize the potential of VL literacy to support sustained viral suppression, we propose a model of psychosocial support, focused on young people's five domains of wellbeing [44], that assumes adherence is precarious and likely to fluctuate. Viral suppression needs to be seen as a journey, recognizing the unstable terrain of adolescents’ lives and not assuming adherence behaviours and VL will remain stable or consistent. Given this, AYPLHIV are likely to need continual support over time, understanding of their individual circumstances and transparent acknowledgement that maintaining adherence is difficult [21, 40, 53]. Improving VL literacy equips adolescents with the knowledge that will allow them to understand their clinical results as part of this journey [36]. However, educating adolescents on the clinical implications of VL results alone does not go far enough. Sustained adherence requires making space for AYPLHIV to share in a supportive environment, for example with their peers, their experiences of managing treatment and to realize with them what viral suppression might mean for their relationships, their physical and mental health, and their personal goals [26, 54].

A first step is to move away from the dichotomized way in which adherence is evaluated on the basis of VL results. If we use the analogy of a traffic light, VL results produce either a green or red light: a go or stop. Reimagining adherence more realistically and compassionately as “always on amber” recognizes that all adolescents are always at risk; that their current location is always only temporary. An amber approach expects there to be problems and invites anticipatory conversations with adolescents about the complicated realities that impact their ability to maintain motivation and routine.

### 4.3 U = U: you cannot become what you do not know is possible

Among population groups where U = U messaging has been promoted, a reduction in stigma and improved self‐image has been reported [35, 55, 56, 57, 58]. Communicating U = U requires crafting the message to respond to different populations’ attitudes, existing knowledge and local contexts [59]. Responsible reception of U = U messaging for adolescents can be introduced by first building VL literacy, and then extended through age‐appropriate approaches, which are in step with AYPLHIVs’ concerns and priorities. Embedding U = U within broader VL literacy may help to alleviate carer and HCW concerns about complacency and the possibility of inconsistency in routine testing, but it also lessens the risk that adolescents hear U = U without a full appreciation of how ART works to make that possible.

### 4.4 Strengths and limitations

The rich insights afforded by in‐depth interviews with young people and the context‐specific outputs produced as a result do have the potential to be conceptually generalizable and, therefore, be adapted to districts in Zimbabwe outside those included in this study, and to countries within the region. All AYPLHIV participants were already engaged in support groups and accessing HIV treatment at clinics where peer‐counsellors are operating, suggesting our findings likely reflect a best‐case scenario. Participants in this study echoed findings in other studies, particularly in terms of the social barriers they experience when navigating their HIV status and treatment adherence within their friendships and intimate relationships.

## 5 CONCLUSIONS

We argue that investing in VL literacy holds great promise and ought to extend beyond simply inviting AYPLHIV to understand how ART is suppressing the virus in their body. It is cogent that we use VL literacy to develop a range of motivating narratives about what a suppressed VL represents in individuals’ social and relational lives, both present and future. Translating the clinical benefits of viral suppression into meaningful narratives relevant to adolescents’ wellbeing and priority concerns may instil the hope and motivation critical to the pursuit of sustained long‐term viral suppression.

## COMPETING INTERESTS

The authors declare that they have no competing interests.

## AUTHORS’ CONTRIBUTIONS

ZMN and SB performed the research. ZMN, SB and FC designed the research study. BS and NW contributed essential input to the design and focus of the study and paper. SB, JL and ZMN analysed the data. SB and JL wrote the first draft of the paper, with all of the co‐authors reviewing subsequent drafts and approving the submitted version.

## Data Availability

The data that support the findings of this study are available on request from the corresponding author. The data are not publicly available due to privacy or ethical restrictions.
